# Foliar applications of a vegetal-derived protein hydrolysate alone or in combination with calcium alleviate heat stress in lettuce by boosting leaf antioxidant activity

**DOI:** 10.3389/fpls.2026.1773490

**Published:** 2026-07-01

**Authors:** Leonardo Fiore, Mariateresa Cardarelli, Simona Proietti, Stefano Moscatello, Giuseppe Colla

**Affiliations:** 1 Department of Agriculture and Forest Science, University of Tuscia, Viterbo, Italy; 2 Istituto di Ricerca sugli Ecosistemi Terrestri, National Research Council (CNR), Porano, Italy; 3 Arcadia Società a Responsabilità Limitata, Rivoli Veronese, Italy

**Keywords:** biostimulant, climate change, high temperature, *Lactuca sativa* L., phenotyping

## Abstract

**Introduction:**

The increase in temperatures caused by climate change represents a major abiotic stress factor negatively affecting yield and quality traits of leafy vegetable crops. The use of protein hydrolysate-based products has been recently proposed as a tool to mitigate the negative effects of heat stress on several crops.

**Methods:**

Multiple foliar applications of a vegetal derived protein hydrolysate alone (PH) or enriched with calcium (Ca-PH) were tested, together with an untreated control, on lettuce plants subjected to a heat stress event (43/30 °C day/night for 3 consecutive days). Morpho-physiological traits were monitored over the trial through a phenotyping platform and a Multi Pigment Meter along with destructive measurements of plant biomass and biochemical and mineral analysis of leaf tissues.

**Results:**

Both protein hydrolysate-based products mitigated heat stress by enhancing fresh weight and dry weight of lettuce shoots in comparison with control treatment. Moreover, both protein-hydrolysate-based products promoted plant uptake of key nutrients like Ca, P, and Mn and the antioxidant activity (FRAP and DPPH) in lettuce leaves. Ca-PH was more effective than PH in promoting a fast recovery of lettuce plants (Digital Biomass) after heat stress as a result of a pre-stress accumulation of proline.

**Conclusion:**

In conclusion, foliar applications of a vegetal-derived protein hydrolysate alone or in combination with Ca can be considered a sustainable approach to counteract the negative effects of heat stress induced by climate change in lettuce crops.

## 1 Introduction

The increase in global temperatures is causing a rise in heat stress events in Mediterranean Countries negatively affecting crop productivity and quality worldwide (Lindsey and Dahlman, 2025). Heat stress leads to the overproduction of reactive oxygen species (ROS) in plant cells, causing oxidative damage, peroxidation of membrane lipids and pigments, loss of membrane permeability, reduced cell membrane thermostability, and chlorophyll degradation, all of which ultimately reduce photosynthetic activity (Oyebamiji et al., 2023). Other consequences include stomatal closure, reduces CO_2_ availability, and damage cellular structures, while suppressing protein synthesis and heat shock protein expression, altering hormonal homeostasis, and decreasing the production of phytohormones and antioxidants (dos Santos et al., 2022; Oyebamiji et al., 2025). Furthermore, heat stress impacts on carbohydrate metabolism by inhibiting key enzymes responsible for starch and sucrose synthesis, reducing carbohydrate availability for plants growth and development (Oyebamiji et al., 2023).

In this scenario, it is important to develop strategies to improve plant tolerance to heat stress, especially in the highly sensitive cool-season crops like lettuce.

Lettuce is one of the most important leafy vegetable for human consumption where growth, yield and quality are severely impaired when temperature exceed 30 °C (Pereira et al., 2024; Duan et al., 2025). Hawrylak-Nowak et al. (2018) reported that lettuce plants grown under heat stress conditions (35/22 °C day/night) showed a reduction in fresh and dry weight of shoots by 47% and 29%, respectively, in comparison with plants grown under optimal temperature regime (22/19 °C day/night). Similarly, in the study conducted by Sun et al. (2022), the authors observed a reduction in fresh and dry weight (33% and 31%, respectively) of shoots in lettuce plants subjected to heat stress (35/30 °C day/night) compared to lettuce plants grown under optimal temperature conditions (22/17 °C day/night).

Heat stress can also affect quality traits of lettuce such as chlorophyll content of leaves. Shin and Lee (2025) exposing lettuce plants for ten consecutive days to high temperature (40/36 °C day/night), documented a significant reduction of chlorophyll *a* and total chlorophyll in leaves compared to control plants (24/20 °C day/night). Similarly, Wang et al. (2021) obtained a reduction in chlorophyll content and total soluble sugars of leaves when plants grown under heat stress (35 and 45 °C) in comparison to control (25 °C). Heat stress can also affect negatively other quality traits of shoots such as leaf shape, internode elongation, rib discoloration, leaf tip burn, premature bolting and ribbiness, all of which reduce the commercial value of lettuce (Pereira et al., 2024).

Plant biostimulants could be a useful tool to enhance crop resistance to heat stress as reported by Carillo (2025). Amino acid-based product was effective in enhancing heat stress tolerance in crops as reported by Botta (2012). In this study, *Lolium perenne* L. was foliarly treated or un-treated with an amino acid-based product and subjected to heat stress (28 and 36 °C); results indicated a higher photosynthetic efficiency (Fv/Fm) in plants treated with amino acids compared to control; moreover, amino-acid application induced an increase in total chlorophyll and carotenoids in leaves that grown at 36 °C. Among the different amino acids, glycine seems to be the most effective amino acid in mitigating heat stress in plants, as reported by Quan et al. (2022): seedlings of Chinese cabbage treated with glycine and exposed at heat stress conditions (45/35 °C 18/8 h day/night for 5 days) showed an increase in fresh and dry weight of shoots by 26 and 36%, respectively, in comparison with un-treated control. Moreover, the authors recorded an increase in chlorophyll fluorescence parameters and antioxidant enzyme activity in seedlings of Chinese cabbage treated with glycine in comparison with un-treated control.

Similarly to amino acid-based products, protein hydrolysates can be also used to mitigate the effects of heat stress on plants, as reported by Francesca et al. (2020): using protein hydrolysate on tomato plants grown at elevated temperatures (up 42 °C), the authors recorded an increase in final yield, number of fruits and antioxidant contents in leaves and fruits in plants treated in comparison with un-treated plants. The above findings demonstrated that protein hydrolysates and amino acid-based products help to mitigate the heat stress in crops through the capacity of these products to induce plant defense responses. Despite these studies, the use of protein hydrolysates to mitigate the effects of heat stress on lettuce is still unexplored. Bioactive compounds of protein hydrolysates (e.g. peptides) may reduce heat stress in plants by stimulating antioxidant defense system of cells and improving plant uptake of crucial nutrients like calcium (Hossain et al., 2024). Moreover, Caruso et al. (2019) and Cristofano et al. (2021) reported an increase of Ca concentration of leaves in lettuce and rocket when a vegetal-derived protein hydrolysate was foliarly sprayed multiple times.

Calcium has a dual role in plants: structurally, Ca contribute to the stability of the cell walls and membranes, and at the same time, calcium is an important second messenger in numerous physiological and biochemical process, including stress perception and the activation of natural defenses in plant, such as stomal movement, ion homeostasis, antioxidant response, and gene expression (Kumari et al., 2022; Hossain et al., 2024; Zheng et al., 2024). Specifically, when exogenous Ca was applied with foliar application, it primarily enters via stomata and redistributed within the cell, allowing rapid and localized increases to support metabolic functions and maintaining cellular homeostasis under both stress and unstress conditions (Feng et al., 2023).

With foliar application of calcium chloride, Ayub et al. (2022) reported an enhancement on fresh and dry weight of shoots and roots, leaf antioxidant enzyme activity and chlorophyll content in tomato seedling subjected to high temperature (42 °C). Moreover, foliar applications of calcium nitrate on spinach grown under heat stress conditions (35/25 °C 14/10 h day/night) resulted in an increase in fresh and dry weight of shoots and roots, chlorophyll and carotenoids contents and in a reduction of antioxidant enzyme activity and electrolyte leakage in comparison to control treatment (uz Zaman et al., 2022). Moreover, Ca application is also effective in mitigating heat stress when it is used as priming agent of seedlings as reported by Bhatia and Asthir (2014) who demostrated that applications of calcium chloride by soaking wheat germinated seeds two hours before under heat stress (45 °C) determined an increase in shoot and root length, total free sugars content, α- and β-amylase activity in comparison to control ().

Recently Ca-peptide biochelates have been proposed to combine nutritional (high Ca availability for plant uptake) and biostimulant action (abiotic stress mitigation, improvement of product quality). However, no information is available in scientific literature about the potential benefits of Ca-peptide biochelates in heat stress mitigation and leaf quality improvement of lettuce crops. Starting from the above considerations, a trial was conducted to evaluate the effects of a vegetal-derived protein hydrolysate (PH) and a vegetal-derived protein hydrolysate combined with calcium (Ca-PH) on lettuce yield and quality traits, before and after heat stress event. Moreover, physiological traits of lettuce leaves were also determined for a comprehensive understanding of treatment effects.

## 2 Materials and methods

### 2.1 Plant material and growth conditions

The trial was conducted in summer 2024 in climate-controlled chamber (Fratelli Della Marca, Environment Makers, LFC1500SXPRO, Rome, Italy) at the Department of Agriculture and Forest Science (University of Tuscia, Viterbo, Italy). Lettuce seeds (*Lactuca sativa* L. cv. Salanova Aquino; Rijk Zwaan, Bologna, Italy) were sown in polystyrene trays filled with commercial substrate containing mainly peat moss (Brill, Georgsdorf, Germany) and located in greenhouse (average germination temperature of 23 °C). When the plant reached fifth-sixth true leaf, they were transplanted into pots (6.5 × 8.5 cm and 10 cm in diameter) filled with 0.65 L with a mixture of field soil and pure sand (50-50%, v/v).

On the 8^th^ Days After Transplanting (DAT), lettuce plants were moved into climate-controlled chamber, with following climate conditions: 60% relative humidity, photosynthetically active radiation (PAR) of 216 µmol m^-2^ s^-1^, 16/8 h day/night at 26/16 °C.

Heat stress period at 43/30 °C day/night was for three days (from 22 to 24 DAT) as used by Collado-Gonzalez et al. (2022). We also included an acclimatation period of two days (from 20 to 21 DAT) before heat stress period where air temperature gradually increased to 32/21 °C day/night on the 20^th^ DAT and to 38/26 °C day/night on 21^st^ DAT. The acclimation period was used to avoid a heat shock to lettuce plants due to the sudden change of air temperature from 26/16 °C to 43/30 °C.

During the heat stress period, the climate conditions was: 60% relative humidity, photosynthetically active radiation (PAR) of 216 µmol m^-2^ s^-1^, 16/8 h day/night at 43/30 °C. After the heat stress period (24^th^ DAT), the initial temperature values were then restored, namely 26/16 °C day/night. All plants were sub-irrigated daily with 100 mL of the following nutrient solution (mg/L): 722.58 Ca(NO_3_)_2_, 136.54 KH_2_PO_4_, 230.39 K_2_SO_4_, 83.58 KNO_3_, 250.00 MgSO_4_, 107.69 NH_4_NO_3_. Micronutrients were added as 24 mg/L of a commercial fertilizer (Mikrom; Cifo S.p.A., Bologna, Italy) containing (g/kg): 5 B, 5 Cu, 40 Fe, 40 Mn, 2 Mo, 10 Zn. Mikrom also provided small amounts of Mg (0.43 mg/L) and S (0.58 mg/L).

### 2.2 Biostimulant characteristics and treatments

A vegetal-derived protein hydrolysate Trainer^®^ (PH) obtained through enzymatic hydrolysis of legume seeds (310 g/kg of peptides and free amino acids) was evaluated together with the vegetal-derived protein hydrolysate combined with calcium CaNOVA^®^ (PH-Ca) (42.9 g/kg of Ca). The protein hydrolysate’s detailed aminogram and other components were reported in detail by Rouphael et al. (2017) and Paul et al. (2019).

Both protein hydrolysate-based products were provided by Hello Nature^®^ (Rivoli Veronese, Italy).

At 8^th^, 15^th^ and 25^th^ DAT the foliar treatments were performed for both products at 4 mL/L, and for control with distillated water. Treatments were arranged in a randomized block design with eight replicates for treatment.

### 2.3 Non-destructive physiological measures and phenotyping

During the trial, before (19^th^ DAT), during (23^rd^ DAT) and after (26^th^ and 29^th^ DAT) heat stress, a non-destructive assessment was performed to determine chlorophylls (transmittance ratio T850/T720), flavonols (transmittance ratio F660/F325), and anthocyanin content (transmittance ratio F660/F525) in lettuce leaves using the Multi-Pigment-Meter (ADC BioScientific Ltd., Hoddesdon, UK). The same instrument automatically calculated the Nitrogen–Flavonol Index (NFI) as the ratio between the chlorophyll and flavonol content. Three leaf measurements were taken for each plant with a total of 24 replicates per treatment (8 plants × 3 leaf measurements).

In addition, on the same dates, the maximum quantum efficiency of photosystem II (Fv/Fm) was measured with FluorPen FP-110 instruments (Photon Systems Instruments spol. Sro, Czech Republic) after 30 minutes of dark adaption of the leaves. One leaf measurement was taken for each plant with a total of 8 replicates per treatment.

Lettuce morphological traits were monitored with a high throughput phenotyping platform (Phenospex, Heerlen, The Netherlands), belonging to Arcadia Spin-off Company (Rivoli Veronese, Italy) and located at the Experimental Farm of Tuscia University.

Before (19^th^ DAT), during (23^rd^ DAT) and after (26^th^ and 29^th^ DAT) heat stress, the plants were transferred from the climate chamber to the phenotyping platform, and two sensors (PlantEye F500 multispectral 3D laser scanners) were used to generate point clouds of plant morphology. In each phenotyping day, eight plants were individually scanned for each treatment.

Two morphological traits were evaluated using multispectral 3D laser scanners: 3D leaf area, and plant height. Digital biomass was automatically calculated by the HortControl software (Phenospex, Heerlen, The Netherlands) by multiplying the plant’s 3D leaf area by its plant height. This mathematical operation gave a measurement of the plant canopy’s volume, which serves as a highly accurate proxy for actual shoot biomass.

### 2.4 Plant biomass determination and mineral analysis

On the 29^th^ DAT, eight lettuce plants per treatments were individually harvested to record the fresh weight of shoots. Shoots dry weight was determined after oven-drying plant tissue at 65 °C until the sample weight remained constant. Dry leaf samples were ground separately in a Wiley mill to pass through a 20-mesh screen.

Leaf mineral analysis was performed on eight dry leaf samples per treatment. Nitrogen concentration was determined using Kjeldahl method (Bremner, 1965): 0.25 g of dry sample was mineralized with sulfuric acid, distillation and titration with 0.1 N HCl. Regarding other elements (P, K, Ca, Mg, Fe, Mn, Zn and Cu), at 0.2 g of dry samples was added with 7.5 mL of 68% HNO_3_ and incubated for 1 h at room temperature. Then, 0.5 mL of 37% HCl (v/v) was added, and the mixture was further incubated for 1 h at room temperature. Finally, 2 mL of 30% H_2_O_2_ was added, and the samples were kept for 30 min before acid mineralization in a microwave oven (180 °C for 15 min). After digestion, the solutions were cooled and diluted with Milli-Q water to a final volume of 15 mL. The samples were analyzed by inductively coupled plasma optical emission spectrometry (ICP-OES, Perkin Elmer^®^ Optima™ 8000 DV) equipped with a CETAC U5000AT ultrasonic nebulizer. The concentration of trace elements was determined using 7 calibration standards (standard solutions, CaPurAn, CPAchem, Stara Zagora, Bulgaria). The wavelengths used for the quantification were as follows: Zn (206,200 nm), Mg (279,079 nm), Ca (317,933 nm), Cu (324,752 nm), Mn (257,610 nm), Fe (259,939 nm), P (213,618 nm) e K (766,491 nm).

### 2.5 Total phenols, flavonoids, and antioxidant activity

Before (19^th^ DAT) and after (26^th^ and 29^th^ DAT) heat stress, four leaves were harvested and frozen with liquid nitrogen and stored at −80 °C. Subsequently, the sample was ground with liquid nitrogen, and an aliquot of 0.2 g was used for analysis of total phenols, flavonoids and antioxidant activity.

For total phenols and flavonoids, extraction was performed using 80% ethanol (pH 2), while for the ferric reducing antioxidant power assay (FRAP) and 2,2-diphenyl-lpicrylhydrazyl assay (DPPH), extraction was carried out with 80% methanol. Both extractions were conducted with 0.2 g of frozen samples and 4 mL of extracting solution, homogenization, and incubation for 1 h at room temperature. The extract was then centrifuged at 4,000 g for 15 min (D3024R, Vetro Scientifica srl, Roma, Italy), and the supernatant was stored at −20 °C.

The total phenols content (TPC) was determined using the Folin–Ciocalteu method (Singleton and Rossi, 1965) with gallic acid (GAE) as a standard. Briefly, 0.2 mL of ethanolic extract was added with 1 mL of Folin–Ciocalteau’s reagent diluted (1:5, v:v) and 0.8 mL of 7.5% sodium carbonate (w/v). The absorbance of the solution was measured after 1 h at 765 nm, and the result was expressed as mM GAE for 100 g of fresh weight.

The flavonoid contents were determined using the colorimetric method (Zhishen et al., 1999) with quercetin (QE) as a standard. Briefly, 0.7 mL of ethanolic extract was added with 0.15 mL of 5% (w/v) sodium nitrite, 0.15 mL of 10% (w/v) aluminum trichloride, and 1 mL of 1 M sodium hydroxide. The absorbance of the solution was measured at 510 nm, and the result was expressed as mM QE for 100 g of fresh weight.

The FRAP assay (Benzie and Strain, 1996) was conducted using 0.03 mL of methanolic extract, 0.09 mL of distilled water, and 0.9 mL of FRAP working solution, consisting of acetate buffer 300 mM (pH 3.6), 2,4,6-tris(2-pyridyl)-s-triazine 10 mM, and FeCl_3_ ∗ 6 H_2_O 20 Mm (10:1:1; v/v/v).

After incubation at 37 °C for 30 min in the dark, the absorbance was read at 593 nm and, through curve calibration with Trolox, the results were expressed as mM Trolox for 100 g^−1^ of fresh weight.

The DPPH assay was conducted using the Brand-Williams et al. method (Brand-Williams et al., 1995). A 0.1 mL of sample was incubated with 0.1 mL of distilled water and 0.8 mL of DPPH 75 μM working solution. After 30 min in the dark, the absorbance was read at 517 nm and the results were expressed as mM Trolox for 100 g^−1^ of fresh weight.

For all these determinations, the Helios Beta Spectrophotometer (Thermo Electron Corporation, Altrincham, UK) was used.

### 2.6 Proline concentration

Proline content in the lettuce leaves (4 leaves per treatment) was determined using the colorimetric method (Bates, 1973). Specifically, 0.2 g of frozen sample was added to 4 mL sulfosalycilic acid 3%, homogenized and centrifuged at 4000 g for 10 minutes at 4 °C (D3024R, Vetro Scientifica). Subsequently, 0.5 mL of supernatant was added with 0.5 mL acid ninidrine solution (containing 2.5% w/v ninhidrine, 60% v/v acetic acid and 40% v/v of 6 M ortophosphoric acid) and 0.5 mL of acetic acid. The samples were incubated for 1 h at 100 °C, and after cooling, 1 mL of toluene was added, homogenized and when two layers were separated, the upper layer was transferred into cuvette and read at 520 nm with Helios Beta Spectrophotometer (Thermo Electron Corporation, Altrincham, UK) and, through curve calibration with proline standards, the results were expressed as µmol g^−1^ of fresh weight.

### 2.7 Carbohydrate profile

Non-structural carbohydrates (NSCs) in lettuce leaves (4 leaves per treatment) were quantified using 10 mg lyophilized powder extracted in 50% ethanol/50% water at 80 °C for 45 min under continuous shaking. After centrifugation (16,000 × g for 5 minutes), the supernatant containing soluble sugars (glucose, fructose, and sucrose) recovered, while starch remained in the pellet. The pellet was washed four times with 50 mM Na acetate buffer (pH 4.5), suspended and autoclaved at 120 °C for 45 min in 1 mL of the same buffer. Samples were then incubated at 50 °C for 1 h with amyloglucosidase (70 U) and α-amylase (4 U) to hydrolyze the starch to glucose. A spectrophotometric coupled enzymatic assay measured the glucose produced by starch hydrolysis.

The supernatant was filtered through a nylon 0.2 µm PPII syringe filter (Whatman Inc., Maidstone, UK), then analyzed through high-performance anion exchange chromatography with pulsed amperometric detection (HPAEC-PAD). An ICS-6000 system equipped with a dual eluent generator cartridge (EGC) module, a CarboPac™ PA200 analytical column (1×250 mm), and a corresponding guard column (1×50 mm) (Thermo Fisher Scientific Dionex™, Waltham, MA, USA) was used. Runs were performed at 30 °C with a 0.063 mL/min flow rate, applying a potassium hydroxide gradient as follows: 17 mM (0–3.5 min), 21 mM (3.5–11 min), 180 mM (11–23 min), and re-equilibration at 17 mM (23–45 min). Quantification of sucrose, glucose, and fructose was achieved using calibration curves generated from HPLC-grade carbohydrate standards (Sigma, Steinheim, Germany), with fucose serving as the internal standard.

### 2.8 Statistical analysis

All data were subjected to analysis of variance (ANOVA). Before analysis, the experimental data set was checked for normal distribution and homogeneity of variance using Levene’s test. Tukey’s test was carried out at p = 0.05 on each of the significant variables measured. For each treatment, linear regression analysis was performed for Digital Biomass and for 3D Leaf Area after heat stress event to evaluate the recovery effect of protein hydrolysate based products. Bivariate Pearson correlation was used to quantify the strength and direction of the linear association between shoot fresh biomass or shoot dry biomass and each mineral nutrient in leaf tissues. The RStudio software version 4.4.2 (RStudio Team, Vienna, Austria) was used for statistical analysis.

## 3 Results

### 3.1 Spectral indices of leaves

In **Table 1**, the results of spectral leaves measurements obtained with the Multi-Pigment Meter are reported. Regarding the chlorophylls content, no significant differences were observed before (19^th^, Days After Transplanting – DAT), during (23^rd^ DAT) and after (29^th^ DAT) heat stress; moreover, at 26^th^ DAT, treatment with protein hydrolysate combined with calcium (Ca-PH) provided the highest value in chlorophylls content, while treatment with protein hydrolysate (PH) gave the lowest value.

**Table 1:** Effect of foliar applications of protein-hydrolysate-based products on multi-pigment readings (chlorophylls, flavonols, anthocyanin and Nitrogen Flavonol Index) of lettuce leaves before (19 DAT), during (23 DAT) and after (26 and 29 DAT) heat stress.

Treatments	19 DAT	23 DAT	26 DAT	29 DAT
Chlorophylls				
Control	0.321 ± 0.057	0.435 ± 0.071	0.426 ± 0.083 ab	0.426 ± 0.082
PH	0.313 ± 0.067	0.422 ± 0.074	0.374 ± 0.075 b	0.408 ± 0.099
Ca-PH	0.310 ± 0.058	0.453 ± 0.062	0.432 ± 0.084 a	0.425 ± 0.097
Significance	ns	ns	*	ns
Flavonols				
Control	0.222 ± 0.033	0.164 ± 0.041	0.166 ± 0.040	0.183 ± 0.042
PH	0.218 ± 0.045	0.185 ± 0.052	0.186 ± 0.053	0.190 ± 0.041
Ca-PH	0.199 ± 0.029	0.161 ± 0.038	0.183 ± 0.036	0.210 ± 0.046
Significance	ns	ns	ns	ns
Anthocyanin				
Control	0.044 ± 0.005	0.027 ± 0.005	0.025 ± 0.004 b	0.021 ± 0.003
PH	0.048 ± 0.005	0.033 ± 0.005	0.043 ± 0.006 a	0.031 ± 0.005
Ca-PH	0.047 ± 0.003	0.028 ± 0.004	0.026 ± 0.004 b	0.020 ± 0.003
Significance	ns	ns	*	ns
Nitrogen flavonol index				
Control	1.467 ± 0.051	2.990 ± 0.218	2.852 ± 0.189 a	2.410 ± 0.146
PH	1.424 ± 0.082	2.614 ± 0.164	2.079 ± 0.116 b	2.234 ± 0.149
Ca-PH	1.532 ± 0.071	2.966 ± 0.170	2.436 ± 0.122 ab	2.113 ± 0.138
Significance	ns	ns	**	ns

PH, protein hydrolysate ‘Trainer^®^’, Ca-PH, protein hydrolysate combined with calcium ‘CaNOVA^®^’. DAT, days after transplanting. ns, *, **, nonsignificant or significant at p ≤ 0.05 and 0.01. Different letters within each column indicate significant differences according to Tukey’s test for p = 0.05, respectively. All data are expressed as mean ± standard error.

No significant differences were observed before (19^th^ DAT), during (23^rd^ DAT) and after (26^th^ and 29^th^ DAT) heat stress. Foliar treatment with PH increased the anthocyanin content after heat stress (26^th^ DAT), while treatment with Ca-PH gave a similar value to control treatment. For Nitrogen Flavonol Index (NFI), no significant differences were observed between control and PH; moreover, at 26^th^ DAT, the control gave the highest value, while PH treatment recorded the lowest value. For anthocyanin and NFI, no significant differences were observed at 19^th^, 23^rd^ and 29^th^ DAT.

No significant differences were observed before (19^th^ DAT), during (23^rd^ DAT) and after (26^th^ and 29^th^ DAT) heat stress regarding the maximum quantum efficiency of photosystem II (Fv/Fm) in lettuce leaves (**Supplementary Table 1**).

### 3.2 Digital morphological traits

**Figure 1:**
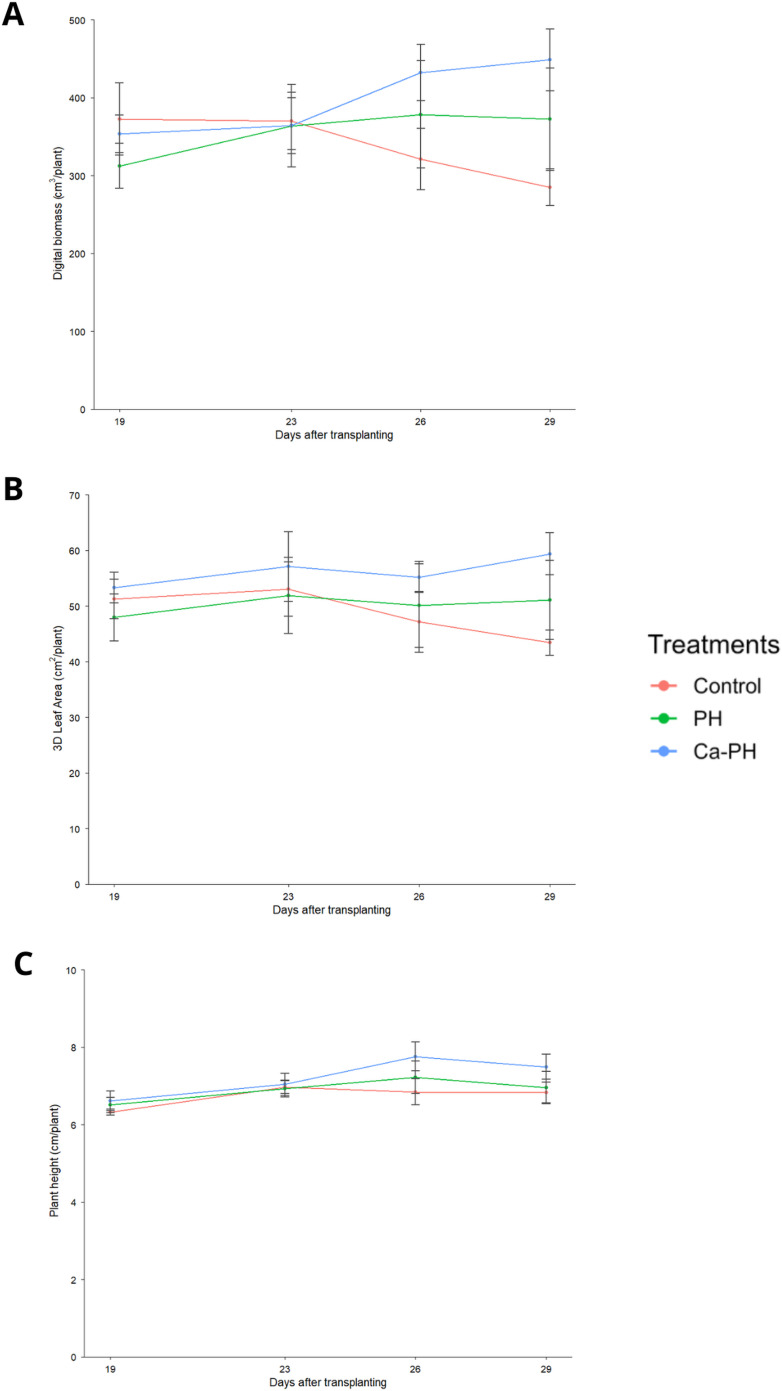
Digital biomass **(A)**, 3D leaf area **(B)**, plant height **(C)** of lettuce plants from HortControl software before (19 DAT), during (23 DAT) and after (26 and 29 DAT) heat stress event. The x axis represents the days in which the scan was made. PH corresponds to ‘Trainer^®^’ while Ca-PH corresponds to ‘Ca-NOVA^®^’. Bars indicate standard errors of the means.


The results obtained by phenotyping platform are reported in **Figure 1**. Digital Biomass, 3D Leaf Area and Plant Height were similar among treatments before heat stress (19^th^ Days After Transplanting, DAT) and during heat stress event (23^rd^ DAT). From heat stress (23^rd^ DAT) to the end of the trial (29^th^ DAT), Digital Biomass (C1) and 3D Leaf Area (C2) linearly declined in control treatment according to the following regressions:

$$y_{C1}=\;-14.16\;x+693.65\;\left(r^{2}=0.99\right)$$

$$y_{C2}=\;-1.60\;x+89.53\;\left(r^{2}=0.98\right)$$

where x are the days after transplanting following the stress event.

No significant changes were observed for Digital Biomass, and 3D Leaf Area in PH treatment from 23^rd^ to 29^th^ DAT while Ca-PH increased Digital Biomass (Ca-PH1) of lettuce after heat stress event as follow:


$$y_{Ca-PH1}=\;14.06\;x+49.22\;\left(r^{2}=0.89\right)$$

where x are the days after transplanting following the stress event.

### 3.3 Shoot biomass

Fresh and dry weight, and dry matter of lettuce shoots are reported in **Table 2**. No significant differences were recorded in dry matter, while both protein hydrolysate-based treatments significantly affected the fresh and dry weight of lettuce shoots. PH and Ca-PH increased fresh weight of lettuce shoots by 73% and 82%, respectively, in comparison to control. Moreover, PH treatment provided the highest value for dry weight of lettuce shoots, while the control gave the lowest value; intermediate value was recorded in Ca-PH treatment.

**Table 2:** Effect of foliar applications of protein hydrolysate-based products on fresh and dry biomass, and dry matter of lettuce shoots at the end of the trial.

Treatments	Shoots		
	Fresh weight (g/plant)	Dry weight (g/plant)	Dry matter (%)
Control	7.22 ± 0.39 b	0.51 ± 0.02 b	8.82 ± 0.25
PH	12.50 ± 0.34 a	0.72 ± 0.07 a	8.10 ± 0.25
Ca-PH	13.17 ± 1.49 a	0.61 ± 0.06 ab	8.29 ± 0.39
Significance	***	*	ns

PH, protein hydrolysate ‘Trainer^®^’, Ca-PH, protein hydrolysate combined with calcium ‘CaNOVA^®^’. ns, *, ***, nonsignificant or significant at p ≤ 0.05 and 0.001. Different letters within each column indicate significant differences according to Tukey’s test for p = 0.05, respectively. All data are expressed as mean ± standard error.

**Figure 2:**
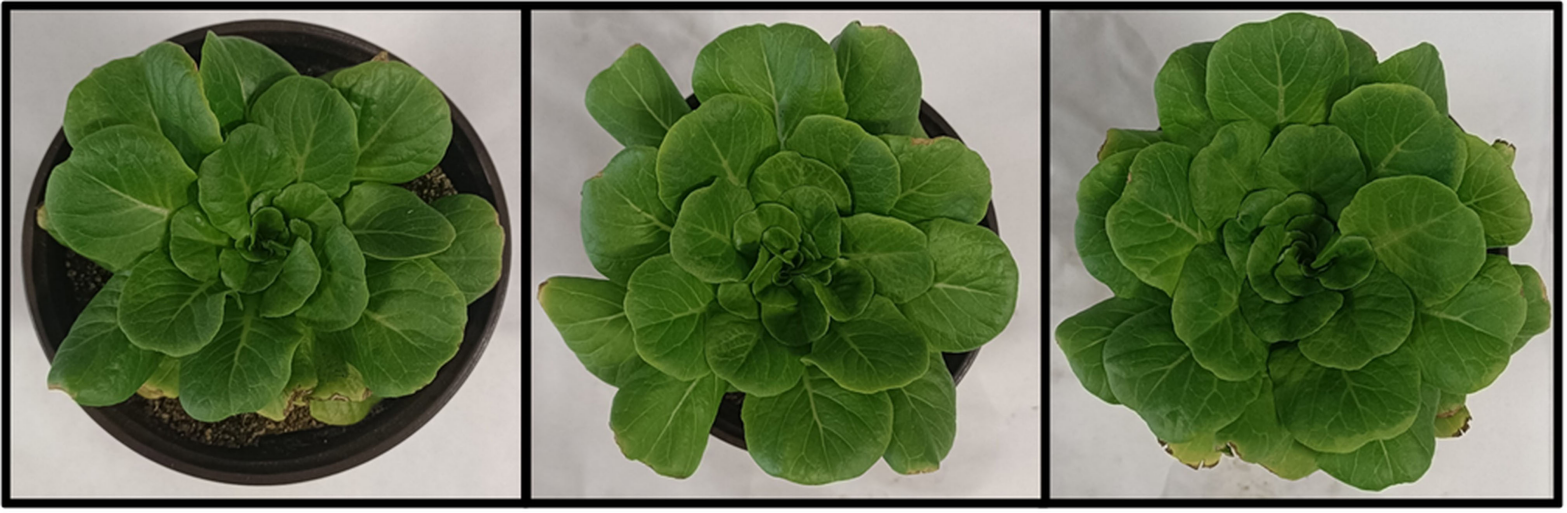
Lettuce plants at the end of trial in Control (left), PH (middle) and Ca-PH (right) treatments.

**Figure 2** shows the visual appearance of lettuce shoots in the three treatments at the end of the trial (29^th^ DAT). In agreement with biomass results, lettuce plants in PH and especially Ca-PH treatment grew beyond the pot edges while control leaves remained inside the pot edges.

### 3.4 Antioxidant activity and phenolic compounds

The results of antioxidants activity (FRAP and DPPH), total phenols content and flavonoids are reported in **Table 3**. Immediately after heat stress (26^th^ Days After Transplanting – DAT) plants treated with protein hydrolysate (PH) significantly enhanced antioxidants activity (FRAP and DPPH) in lettuce leaves respect to un-treated plants, that record the lowest value; intermediate value was obtained in plant treated with protein hydrolysate combined with calcium (Ca-PH). No significant differences among treatments were observed in antioxidant activity (FRAP and DPPH) in lettuce leaves before (19th DAT) and after (29th DAT) heat stress. Total phenols content in lettuce leaves was significantly affected by foliar treatments. Before heat stress (19^th^ DAT), the un-treated plants showed the highest value in total phenols content, while the lowest value was obtained in plants treated with Ca-PH; intermediate value was recorded in PH treatment. After heat stress (26^th^ and 29^th^ DAT), plants treated with PH showed the highest value, while the lowest values were obtained in Ca-PH treatment; intermediate values were observed in un-treated. Before (19th DAT) and after (26th and 29th DAT) heat stress event, the flavonoids content in lettuce leaves did not show significant differences among foliar treatments.

**Table 3:** Effect of foliar applications of protein hydrolysate-based products on ferric reducing antioxidant power assay (FRAP), 2,2-diphenyl-picrylhydrazyl assay (DPPH), total phenols and flavonoids concentration of lettuce leaves before (19 DAT) and after (26 and 29 DAT) heat stress.

Treatment	19 DAT	26 DAT	29 DAT
FRAP (mM Trolox 100 g^-1^ fresh weight)			
Control	456.98 ± 22.88	393.60 ± 13.65 b	606.11 ± 42.54
PH	425.06 ± 29.72	454.59 ± 12.97 a	602.50 ± 30.15
Ca-PH	397.45 ± 10.25	410.10 ± 16.33 ab	530.44 ± 23.98
Significance	ns	*	ns
DPPH (mM Trolox 100 g^-1^ fresh weight)			
Control	313.81 ± 15.34	351.64 ± 15.34 b	425.39 ± 17.02
PH	271.39 ± 16.58	402.45 ± 16.58 a	447.06 ± 16.24
Ca-PH	302.82 ± 10.46	381.51 ± 10.46 ab	423.83 ± 16.31
Significance	ns	*	ns
Total Phenols (mM GAE 100 g^-1^ fresh weight)			
Control	768.87 ± 25.46 a	761.34 ± 24.38 ab	786.56 ± 29.65 ab
PH	722.59 ± 14.04 ab	792.42 ± 36.90 a	841.73 ± 20.40 a
Ca-PH	695.57 ± 14.95 b	688.37 ± 12.85 b	733.77 ± 21.76 b
Significance	*	**	**
Flavonoids (mM QE 100 g^-1^ fresh weight)			
Control	361.70 ± 23.84	319.41 ± 21.80	404.39 ± 21.80
PH	336.05 ± 21.86	322.48 ± 28.92	437.41 ± 28.92
Ca-PH	302.60 ± 20.51	290.88 ± 15.93	290.88 ± 15.93
Significance	ns	ns	ns

PH, protein hydrolysate ‘Trainer^®^’, Ca-PH, protein hydrolysate combined with calcium ‘CaNOVA^®^’. DAT, days after transplanting. ns, *, **, nonsignificant or significant at p ≤ 0.05 and 0.01. Different letters within each column indicate significant differences according to Tukey’s test for p = 0.05, respectively. All data are expressed as mean ± standard error.

### 3.5 Proline

**Figure 3:**
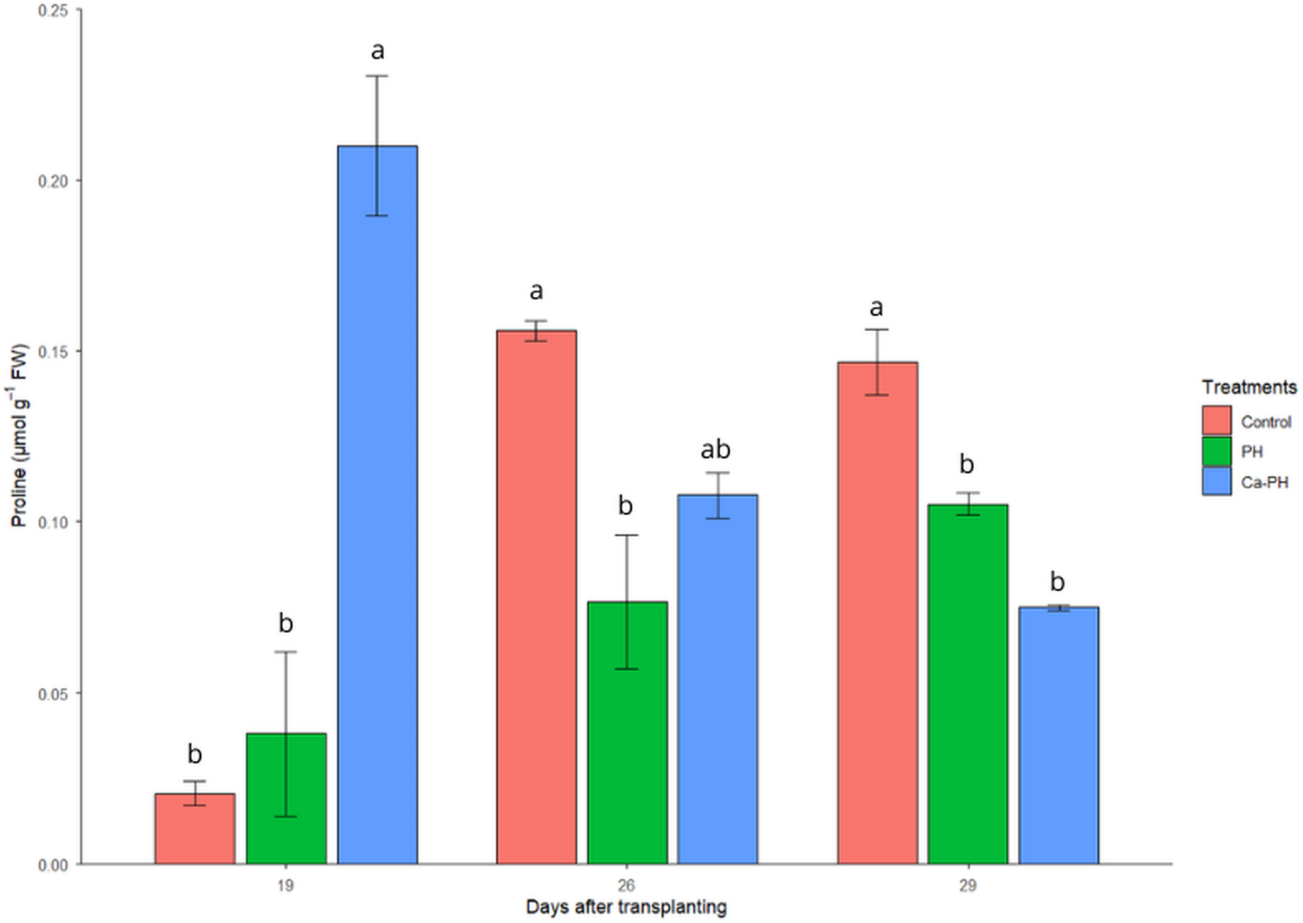
Effects of foliar applications of protein hydrolysate-based products on proline concentration (µmol g^−1^ of fresh weight) of lettuce leaves before (19 DAT) and after (26 and 29 DAT) heat stress [Trainer^®^’ (PH), ‘CaNOVA^®^’ (Ca-PH)]. Different letters within each histogram group indicate significant differences according to Tukey’s test for p = 0.05, respectively. All data are expressed as mean ± error standard.

Proline concentration of lettuce leaves before (19 DAT) and after (26 and 29 DAT) heat stress is shown in **Figure 3**. Before heat stress event (19^th^ DAT), the plants treated with protein hydrolysate combined with calcium (Ca-PH) showed higher value than control and protein hydrolysate (PH) treatments (p< 0.01), while no significant difference was observed between control and PH treatments. Immediately after heat stress (26^th^ DAT), proline level in untreated leaves was higher than PH treatment (p< 0.01), while the Ca-PH treatment recorded intermediate value. Similarly, at the end of trial (29^th^ DAT) the control leaves showed the highest proline level compared to PH and Ca-PH (p< 0.01), while no significant difference was observed between the two-protein hydrolysate-based products.

### 3.6 Mineral profile

Macro- and microelement concentrations in dry leaves are reported in **Tables 4** and **5**, respectively. No significant differences were observed on the leaf N and K concentrations, while significant differences were observed for P, Ca and Mg. Leaf concentrations of P and Mg were highest in PH treatment in comparison with control treatment while leaf Ca concentration was highest in both protein hydrolysate-based treatments.

**Table 4:** Effect of foliar applications of protein hydrolysate-based products on macronutrient concentrations in lettuce leaves at the end of the trial.

Treatment	Macronutrient concentration (g kg^-1^ DW)				
	N	P	K	Ca	Mg
Control	44.20 ± 1.21	0.20 ± 0.04 b	48.8 ± 0.29	27.63 ± 0.54 b	4.71 ± 0.14 b
PH	46.34 ± 1.26	0.31 ± 0.03 a	55.1 ± 0.34	33.82 ± 1.49 a	6.64 ± 0.16 a
Ca-PH	43.68 ± 0.47	0.28 ± 0.04 ab	53.3 ± 0.36	38.31 ± 1.48 a	5.19 ± 0.14 b
Significance	ns	*	ns	***	***

PH, protein hydrolysate ‘Trainer^®^’, Ca-PH, protein hydrolysate combined with calcium ‘CaNOVA^®^’. ns, ***, nonsignificant or significant at p ≤ 0.001. Different letters within each column indicate significant differences according to Tukey’s test for p = 0.05, respectively. All data are expressed as mean ± standard error.

**Table 5:** Effect of foliar applications of protein hydrolysate-based products on micronutrient concentrations in lettuce leaves at the end of the trial.

Treatment	Micronutrient concentration (mg kg^-1^ DW)			
	Fe	Mn	Zn	Cu
Control	319.91 ± 4.501	80.60 ± 3.42 b	48.07 ± 2.03	23.95 ± 1.45 ab
PH	276.13 ± 15.32	115.40 ± 2.73 a	44.83 ± 1.73	28.45 ± 2.71 a
Ca-PH	364.83 ± 34.08	113.37 ± 4.98 a	45.32 ± 0.72	18.93 ± 0.22 b
Significance	ns	***	ns	**

PH, protein hydrolysate ‘Trainer^®^’, Ca-PH, protein hydrolysate combined with calcium ‘CaNOVA^®^’. ns, **, ***, nonsignificant or significant at p ≤ 0.01 and 0.001. Different letters within each column indicate significant differences according to Tukey’s test for p = 0.05, respectively. All data are expressed as mean ± standard error.

No significant differences were observed on the leaf Fe and Zn concentrations, while significant differences were recorded on the leaf Mn and Cu concentration. Leaf Mn concentration was highest in both protein hydrolysate-based treatments while leaf Cu concentration decreased in Ca-PH in comparison with PH; intermediate value of leaf Cu concentration was obtained in control treatment.

Shoot fresh biomass was positively correlated with P (r=0.649*), Ca (r=0.806**) and Mn (r=0.872**) in leaf tissues while shoot dry weight was positively correlated with Ca (r=0.611**), and Mg (r=0.681**) in leaf tissues.

### 3.7 Carbohydrates

Total soluble sugars (fructose, glucose and sucrose) and starch content in lettuce leaves are reported in **Table 6**.

**Table 6:** Effect of foliar applications of protein hydrolysate-based products on carbohydrates content of lettuce leaves before (19 DAT) and after (26 and 29 DAT) heat stress.

Treatment	19 DAT	26 DAT	29 DAT
	mg g^-1^ DW		
Fructose			
Control	24.951 ± 1.569	35.080 ± 4.301	21.880 ± 0.922 b
PH	23.639 ± 1.983	35.991 ± 5.111	32.206 ± 2.721 a
Ca-PH	30.158 ± 1.953	30.186 ± 4.378	34.216 ± 1.735 a
Significance	ns	ns	**
Glucose			
Control	16.703 ± 1.431 b	30.781 ± 3.796	18.112 ± 0.830 b
PH	15.626 ± 1.354 b	31.100 ± 4.856	26.658 ± 2.642 ab
Ca-PH	22.346 ± 1.212 a	27.760 ± 4.538	30.089 ± 2.864 a
Significance	**	ns	*
Sucrose			
Control	23.521 ± 2.371	18.410 ± 2.108	18.061 ± 1.880
PH	21.404 ± 1.809	19.667 ± 2.624	22.028 ± 1.746
Ca-PH	23.749 ± 1.844	18.679 ± 1.352	21.514 ± 3.406
Significance	ns	ns	ns
Total soluble sugars			
Control	64.599 ± 2.875	78.638 ± 8.101	58.756 ± 2.608 b
PH	61.808 ± 5.195	73.371 ± 2.068	75.857 ± 3.895 a
Ca-PH	73.460 ± 3.223	62.607 ± 8.307	82.143 ± 3.545 a
Significance	ns	ns	**
Starch			
Control	5.002 ± 0.291	1.995 ± 0.045	3.845 ± 0.250
PH	4.512 ± 0.497	1.824 ± 0.146	4.115 ± 0.437
Ca-PH	5.147 ± 0.291	1.701 ± 0.159	4.062 ± 0.493
Significance	ns	ns	ns

PH, protein hydrolysate ‘Trainer^®^’, Ca-PH, protein hydrolysate combined with calcium ‘Canova^®^’. ns, *, **, nonsignificant or significant at p ≤ 0.05 and 0.01. Different letters within each column indicate significant differences according to Tukey’s test for p = 0.05, respectively. All data are expressed as mean ± standard error.

Fructose content was significantly affected by treatments only at 29^th^ DAT with highest values in both PH-treated leaves. Glucose content in lettuce leaves before heat stress (19^th^ DAT) was significantly higher in protein hydrolysate combined with calcium (Ca-PH) in comparison to PH and control while no significant differences were recorded at 26^th^ DAT; at 29^th^ DAT, Ca-PH exhibited again the highest glucose content in comparison to control treatment. At the end of trial (29^th^ DAT), total soluble sugars content was significantly higher in both PH-treated leaves then control while no significant differences were recorded on 19^th^ and 26^th^ DAT.

No significant differences were observed in sucrose and starch content of lettuce leaves before (19^th^ Days After Transplanting, DAT) and after (26^th^ and 29^th^ DAT).

## 4 Discussion

With the global rise in temperatures, heat stress events are becoming increasingly frequent in Mediterranean countries, severely affecting yield and quality of crops (Lindsey and Dahlman, 2025). Among climate change, it’s essential to adopt a sustainable strategy to enhance the resistance of crops to high temperatures. Previous studies demonstrated that amino acid-based products (Botta, 2012; Quan et al., 2022) and protein hydrolysate (Francesca et al., 2020) can be used to increase crops resistance to heat stress. In this study, two vegetal protein hydrolysate-based products (alone, PH, or combined with calcium, Ca-PH) mitigated heat stress in lettuce by increasing fresh (73% and 82%, respectively) and dry weight (41% and 20%, respectively) of shoots compared to control. This effect was associated with a rapid recovery of Digital Biomass (**Figure 1A**) compared to control treatment after heat stress especially when protein hydrolysate was enriched with Ca.

The highest final biomass of lettuce shoots observed with protein hydrolysate-based treatments was associated with an increase in fructose, glucose and total soluble carbohydrates (**Table 6**) which can have contributed to mitigate heat stress acting as osmoprotectants, signaling molecules, and carbon sources (Aloryi et al., 2026).

Photosynthesis is among the process most sensitive to elevated temperatures (Mahajan et al., 2025), and several authors have reported a reduction in photosynthetic activity in crops grown under heat stress (Botta, 2012; Niu et al., 2022). In this study, the maximum quantum efficiency of Photosystem II of all treatments was within the optimal range (Maxwell and Johnson, 2000) indicating that the magnitude of heat stress (3 days at 43/30 °C) did not damage the potential for photosynthetic power.

When plants are exposed to abiotic stress, they activate a complex network of defense mechanisms, including antioxidants production, metabolism adjustments, and gene regulation to enhance their stress tolerance (Rao et al., 2025). Among these response, plants accumulate compatible solutes, such as proline, that help to maintain osmotic balance, protect membranes and proteins, and scavenge ROS (Hayat et al., 2012). The increase in proline concentration in lettuce leaves before heat stress with Ca-PH treatment may be associated with the intercellular signal role of calcium, which can induce proline accumulation (Cheng et al., 2013). The accumulation of proline in Ca-PH treated leaves before heat stress could have contributed to mitigating the heat stress damage on plants. Because proline has also been reported as stress marker (Hemadri Reddy et al., 2024), the increase of proline concentration in control leaves after heat stress event (**Figure 3**) indicates that control plants experienced a stronger metabolic stress in comparison to both PH based treatments. Moreover, the decrease in proline concentration after heat stress in both PH based treatments, was accompanied by an increase in antioxidant activity (FRAP and DPPH) of lettuce leaves (**Table 3**).

High temperatures, like other types of abiotic stress, induce the overproduction of ROS in plants, leading to oxidative stress and cellular damage. To mitigate this, plant cells activate enzymatic and non-enzymatic antioxidant defenses (Chaves et al., 2020).

Protein hydrolysate-based products have shown to increase the antioxidant activity in plant tissues (Ciriello et al., 2022; Leporino et al., 2025) by modulating key antioxidant enzymes (Alzate Zuluaga et al., 2023) in vegetable crops under abiotic stress conditions.

In the current study, the increase in antioxidant activity after stress event was not associated with an increase of antioxidant compounds (e.g., total phenols and flavonoids) in lettuce leaves (**Table 3**); therefore, the protein hydrolysate mediated increase in leaf antioxidant activity after heat stress may be caused by an enhancement of antioxidant enzyme activity.

The enzymatic antioxidant defense of plants involves the action of superoxide dismutase (SOD), which converts superoxide radicals (O_2_^-^) into molecular oxygen (O_2_) and hydrogen peroxide (H_2_O_2_). Subsequently, peroxidases (POD) and catalase (CAT) act to complete the detoxification process of ROS (Girma et al., 2022). Mineral elements, in addition to their nutritional role, are also essential cofactors for the synthesis and functioning of antioxidant enzymes (SOD, POD and CAT, Mishra et al., 2023). Mishra et al. (2023) reported that Mn is a cofactor for SOD and converts harmful superoxide radicals into less harmful hydrogen peroxide (H_2_O_2_) and oxygen (O_2_), protecting cells from oxidative stress. Therefore, in the current experiment, the increase of Mn concentration in lettuce leaves (**Table 5**) treated with both protein hydrolysate-based products, may have contribute to increase the SOD activity reducing oxidative stress of lettuce plants.

The increase of Ca concentration in lettuce leaves treated with PH and Ca-PH may have contribute to mitigate heat stress as reported in several crops (Feng et al., 2023; Gupta et al., 2023) where an increase of antioxidant enzyme activities (SOD, CAT and POD) was observed in spinach leaves (uz Zaman et al., 2022), an enhancement in biochemical defense system (DPPH, proline, phenol) was reported in sweet pepper leaves (Motamedi et al., 2019) or an activation of regulatory factors, like calcium-dependent protein kinases (CsCDPK20 and CsCDPK26), involved in the improvement of antioxidant defense as described in tea plants (Gupta et al., 2023). Therefore, the highest Ca concentration recorded in leaves treated with both protein hydrolysate-based products may have mitigate the heat stress damage to cell membrane boosting the antioxidant defense in lettuce leaves. Moreover, P plays a pivotal role in enhancing heat stress tolerance by stabilizing photosynthesis, stimulating antioxidant defenses to contrast oxidative damage, and maintaining cell membrane integrity (Kayoumu et al., 2022). The above findings on leaf P concentration can also explain the improved heat stress tolerance of lettuce plants when they were foliarly treated with both protein hydrolysate-based products. Therefore, the improvement of mineral status associated with PH-based product applications was linked to the enhancement of antioxidant activity and overall heat stress tolerance of lettuce plants.

## 5 Conclusions

This study demonstrated that foliar applications of vegetal-derived protein hydrolysates-based products, alone (PH) or combined with calcium (Ca-PH), can mitigate heat stress in lettuce plants by enhancing the plant uptake of key nutrients like Ca, P, and Mn, the concentration of soluble sugars, and the antioxidant activity (FRAP and DPPH) in lettuce leaves. Ca-PH was more effective than PH in promoting a fast recovery of lettuce plants (Digital Biomass) after heat stress as a result of a pre-stress accumulation of the multifunctional amino acid proline (osmoprotectant, antioxidant, and protein stabilizer). In conclusion, foliar applications of vegetal-derived protein hydrolysate alone or in combination with Ca can be considered a sustainable approach to counteract the negative effects of heat stress induced by climate change in lettuce crops.

## Data Availability

The raw data supporting the conclusions of this article will be made available by the authors, upon reasonable request.
