# Supplementary material for: Foliar applications of a vegetal-derived protein hydrolysate alone or in combination with calcium alleviate heat stress in lettuce by boosting leaf antioxidant activity
Source: Front Plant Sci. 2026 Jul 1;17:1773490. doi: 10.3389/fpls.2026.1773490 (PMC13368515; doi:10.3389/fpls.2026.1773490)
Supplement: Supplementary file 1 [file Table1.docx]

Supplementary Material

# Supplementary Figures and Tables

**Supplementary Table 1.** Effect of foliar applications of protein-hydrolysate based products on the maximum quantum efficiency of photosystem II (Fv/Fm) of lettuce leaves before (12 and 19 DAT), during (23 DAT) and after (26 and 29 DAT) heat stress

| Treatments | 12 DAT | 19 DAT | 23 DAT | 26 DAT | 29 DAT |
| --- | --- | --- | --- | --- | --- |
| Control | 0.859 ± 0.003 | 0.851 ± 0.002 | 0.833 ± 0.007 | 0.848 ± 0.008 | 0.848 ± 0.003 |
| PH | 0.850 ± 0.003 | 0.847 ± 0.003 | 0.827 ± 0.012 | 0.828 ± 0.016 | 0.846 ± 0.009 |
| Ca-PH | 0.857 ± 0.002 | 0.847 ± 0.001 | 0.834 ± 0.007 | 0.852 ± 0.006 | 0.854 ± 0.002 |
| Significance | ns | ns | ns | ns | ns |

PH, protein hydrolysate ‘Trainer®’, Ca-PH, protein hydrolysate combined with calcium ‘CaNOVA®’. DAT, days after transplanting. ns, nonsignificant according to Tukey’s test (p = 0.05). All data are expressed as mean ± standard error.
